# Supplementary material for: Colorimetric Chemosensor Based on Fe3O4 Magnetic Molecularly Imprinted Nanoparticles for Highly Selective and Sensitive Detection of Norfloxacin in Milk
Source: Foods. 2023 Jan 7;12(2):285. doi: 10.3390/foods12020285 (PMC9858306; doi:10.3390/foods12020285)
Supplement: Supplementary file 1 [file foods-12-00285-s001.zip › foods-2103553-supplementary.pdf]

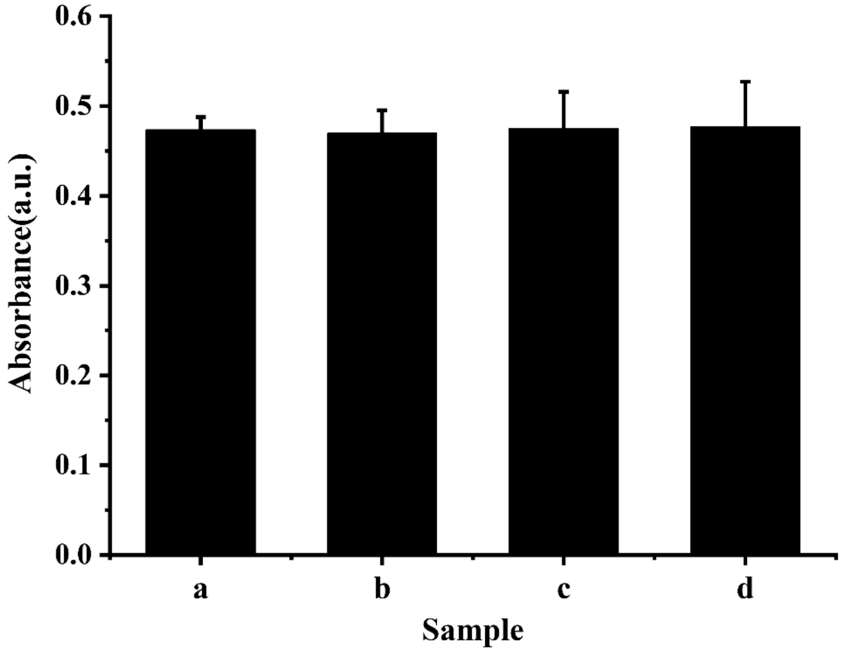

Figure S1. Fe_3O_4 MMIPs NPs colorimetric method for the detection of actual samples (a-d: ultrapure water, milk sample 1, milk sample 2, milk sample 3).

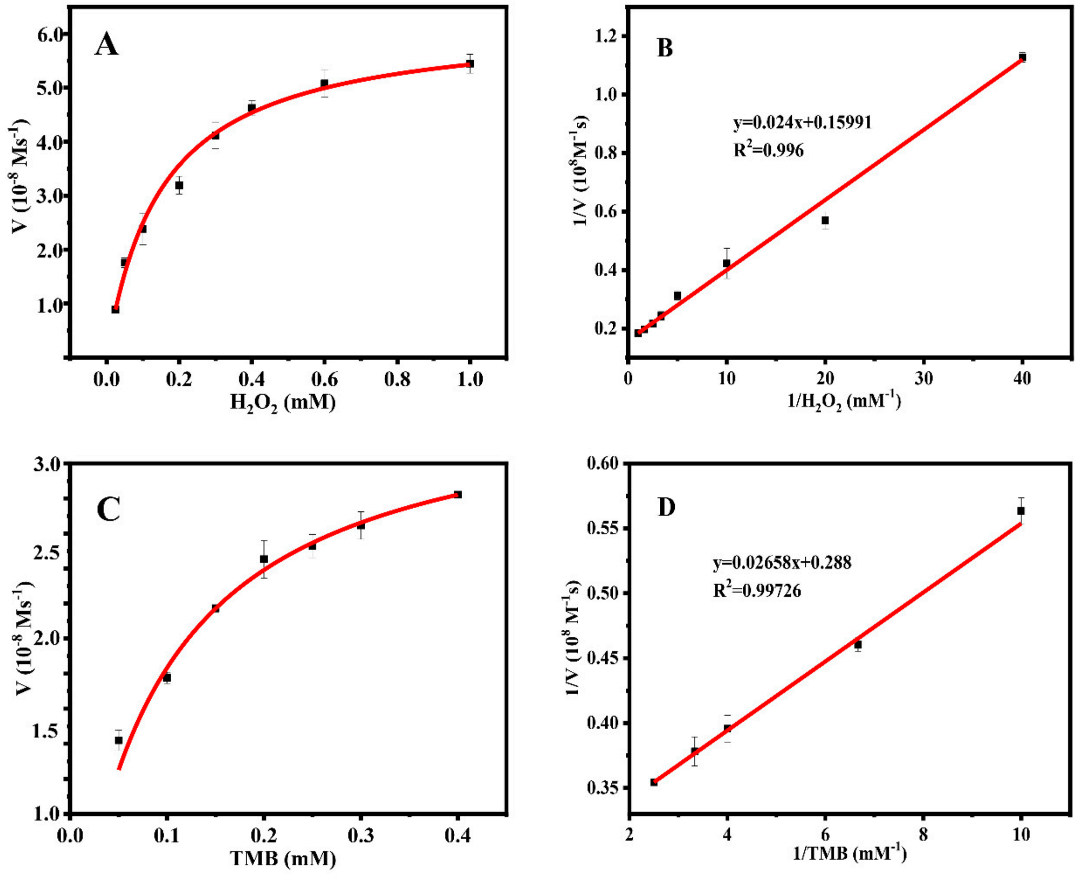

Figure S2. Kinetic analysis of Fe_3O_4 MMIPs NPs' peroxidase-like catalytic activity (A and B: Michaelis-Menten curves and Lineweaver-Burk plot of H_2O_2, C and D: Michaelis-Menten curves and Lineweaver-Burk plot of TMB).
